# National Immunization Program Decision Making Using the CAPACITI Decision-Support Tool: User Feedback from Indonesia and Ethiopia

**DOI:** 10.3390/vaccines12030337

**Published:** 2024-03-20

**Authors:** Maarten Jansen, Dijana Spasenoska, Mardiati Nadjib, Desalegn Ararso, Raymond Hutubessy, Anna-Lea Kahn, Philipp Lambach

**Affiliations:** 1 Immunization, Vaccines and Biologicals Department, World Health Organization, 1202 Geneva, Switzerland; hutubessyr@who.int (R.H.); kahna@who.int (A.-L.K.); lambachp@who.int (P.L.); 2 Department of Social Policy, London School of Economics and Political Science, London WC2A 2AE, UK; d.spasenoska@lse.ac.uk; 3 Department of Health Policy and Administration, Faculty of Public Health, Universitas Indonesia, Depok 16424, Indonesia; mardiatinadjib@gmail.com; 4 Ethiopian Public Health Institute, Addis Ababa 1242, Ethiopia; desalegnararso1@gmail.com

**Keywords:** decision-support, HTA, immunization, MCDA, priority setting, vaccine, new vaccine introduction, LMICs

## Abstract

To ensure that limited domestic resources are invested in the most effective interventions, immunization programs in low- and middle-income countries (LMICs) must prioritize a growing number of new vaccines while considering opportunities to optimize the vaccine portfolio, as well as other components of the health system. There is a strong impetus for immunization decision-making to engage and coordinate various stakeholders across the health system in prioritization. To address this, national immunization program decision-makers in LMICs collaborated with WHO to structure deliberation among stakeholders and document an evidence-based, context-specific, and transparent process for prioritization or selection among multiple vaccination products, services, or strategies. The output of this effort is the Country-led Assessment for Prioritization on Immunization (CAPACITI) decision-support tool, which supports using multiple criteria and stakeholder perspectives to evaluate trade-offs affecting health interventions, taking into account variable data quality. Here, we describe the user feedback from Indonesia and Ethiopia, two initial countries that piloted the CAPACITI decision-support tool, highlighting enabling and constraining factors. Potential immunization program benefits and lessons learned are also summarized for consideration in other settings.

## 1. Introduction

Global recommendations and support from financing and supply agencies, such as Gavi and UNICEF, have historically guided decision-making processes regarding new vaccines to be introduced in low- and middle-income countries (LMICs) [1]. As countries transition to an increase in domestic co-financing and a reduction in donor funding, they must prioritize their investments. In the context of an increasingly large portfolio of vaccine options and the pursuit of disease control targets (e.g., equity, elimination, and eradication) for vaccines already in the portfolio, it is critical that allocation decisions and the inherent trade-offs involved are carefully considered and sensitive to the country-specific context. Moreover, there is an increase in calls to improve coordination of care across settings and sectors, including immunization programs, expanding intersectoral partnerships [2]. The Immunization Agenda 2030 (IA2030) strategy to extend the benefits of vaccines to everyone everywhere is underpinned by four core principles: it puts people in the center, is led by countries, is implemented through broad partnerships, and is driven by data [3]. To ensure country ownership and transparent decision-making in countries, flexible guidance that can be tailored to individual country contexts and varying stakeholders across sectors is needed [1].

Significant progress has been made to enhance the capacity for immunization decision-making in LMICs [4]. Numerous countries have established independent advisory bodies known as national immunization technical advisory groups (NITAGs) to guide policy development [4]. Additionally, an expanding array of tools and information databases are now accessible to facilitate the collection and synthesis of evidence [4]. Beyond these measures, many countries require a robust and credible process for engaging in structured dialogue and interpreting evidence when comparing various options to improve the legitimacy of decision-making [5,6,7].

The Country-led Assessment for Prioritization on Immunization (CAPACITI) decision-support tool was developed by the World Health Organization (WHO) in collaboration with national immunization program decision-makers [1]. It aims to structure deliberation among relevant stakeholders from across the health system and document an evidence-based, context-specific, and transparent process for prioritizing or selecting multiple vaccination products, services, or strategies [1]. Users select the policy or programmatic question that needs to be addressed and can freely determine which options will be compared. This ensures the broad applicability of the tool across pathogens and vaccines and supports both selection between multiple options (product choice, schedule choice, delivery strategy) and ranking of multiple options (new vaccine introduction prioritization, prioritization of vaccine introduction or delivery strategies, or prioritization of immunization and non-immunization alternatives). Botwright et al. (2021) provide an illustrative example based on a CAPACITI pilot country workshop, where three HPV vaccine products were compared against various criteria, in their article entitled ‘*The CAPACITI Decision-Support Tool for National Immunization Programs*’ [1].

This CAPACITI tool is particularly suited for decisions requiring the comparison of two or more health interventions, deliberated by multiple national stakeholders based on evidence across disciplines, taking into account variable data quality [1]. The structure of the tool is informed by best practices in the fields of Health Technology Assessment (HTA) and multi-criteria decision analysis (MCDA) while ensuring practicality [3,7].

Here, we present user-feedback from Indonesia and Ethiopia based on their experiences using the CAPACITI decision-support tool in different ways to respectively develop recommendations for their Ministries of Health (MoH). These countries are among the top countries in the number of children who have never been vaccinated [8]. The national immunization programs from both countries expressed interest in using the tool for decision-making on vaccine introduction and portfolio optimization. The aim of this paper is to offer insights and highlight lessons learned for other countries that may also wish to apply the tool.

## 2. Materials and Methods

### 2.1. The CAPACITI Decision-Support Tool

The CAPACITI decision-support tool (version 2.1) and the underlying methodology have been developed through an iterative approach from 2018 to 2020, in consultation with 13 countries across the WHO regions of Africa, the Americas, Southeast Asia, and the Western Pacific, as well as technical agencies and advisory committees to WHO. The tool’s development and content are well-documented in previous publications [1,6].

The CAPACITI decision-support tool is based on Excel and structured into 5 steps: decision question, criteria for decision making, evidence assessment, appraisal, and recommendation (see Table 1). Implementation of the approach is flexible, allowing end-users to make their own choices, informed by their priorities and program context. The choices consist of what options users would like to consider, which stakeholders to involve, what factors (or criteria for decision-making) are taken into account, how evidence collection is organized, and how the interpretation (or appraisal) of evidence summaries can best be handled. The methodology is adaptable to existing country processes and can follow any type of multi-criteria decision analysis or a hybrid approach. The Excel-based tool guides the user through the 5 steps and allows for documentation of discussions in a transparent manner, with an emphasis on broad stakeholder engagement and country ownership.

The tool offers key considerations and questions under each step, but the fields in each step of the tool are left blank. It is anticipated that certain components will (and should) be prefilled at the country level to tailor to the country context, streamline the process, and ensure consistency and accountability of recommendations. For implementation, it is essential to embed the CAPACITI decision-support tool within the existing decision-making architecture of countries, i.e., using existing recommendation committees when appropriate and feasible. Typically, the committee developing the recommendation convenes on two occasions. The first is to select and agree on the criteria for decision-making, and the second is to conduct the appraisal. While global and regional actors can provide technical support on functionality, it is the countries themselves that choose the decision-making approach that works best for them. The main determinants of time required to complete the process are data collection and analysis requirements, personnel availability, and number of in-person meetings.

### 2.2. Country Implementation

As can be noted from Table 2, Indonesia and Ethiopia each applied the CAPACITI decision-support tool in different manners for different purposes, thereby yielding different results.

#### 2.2.1. Indonesia

The Indonesian Technical Advisory Group for Immunization (ITAGI) implemented the CAPACITI tool with support from their health economics working group, together with the Expanded Program on Immunization (EPI) department at the MoH. The objective was to consider preferred scale-up scenarios for two new vaccine introductions, the Rotavirus and the Human Papillomavirus (HPV) Vaccine, in the context of a rapid nationwide scale-up of Pneumococcal (PCV) vaccination. The vaccine introduction decision was part of the multi-year strategic planning. ITAGI started the decision-support process in October 2019 with the recommended initial in-person consultation to select criteria and outcome measures, assign criteria weights, and develop scoring scales. During this consultation, relevant government stakeholders were engaged, including representatives from various ministries, such as the MoH, Ministry of Planning, and the Ministry of Finance, and various directorates, such as Health Surveillance and Quarantine, Pharmaceutical Services, Public Drug Governance, Public Health, and Nutrition. Key partners were also involved, including the WHO Indonesia country office, UNICEF Indonesia country office, Clinton Health Initiative (CHAI), and the Thinkwell Institute’s Immunization Costing Action Network (ICAN). The selected criteria for decision-making included the burden of disease, impact, vaccine availability, adverse events following immunization (AEFI), cold chain capacity, budget impact, community acceptance, schedule, local production, cost-effectiveness, and level of wastage.

The health economics working group oversaw the evidence assessment for each of the options under consideration and prepared evidence statements and a summary performance matrix as key inputs into the subsequent appraisal step. In November 2021, after facing delays due to the COVID-19 pandemic, a second face-to-face consultation was organized, per CAPACITI’s recommended practices, to appraise the prepared evidence statements and the performance matrix. The CAPACITI decision-support tool allows for the generation of interactive visual aids, which were used to guide and structure discussion by clearly demonstrating the impact of various factors on the total score for each of the options under consideration. The deliberation resulted in a well-documented recommendation, which was presented to the Ministry of Health, along with both the process and the underlying rationale.

#### 2.2.2. Ethiopia

The implementation of the CAPACITI tool in Ethiopia was led by the Ethiopian Public Health Institute (EPHI), bringing together stakeholders and deliberating vaccine portfolio optimization to increase coverage for measles vaccination and identify related evidence needs. The decision question focused on the potential switch from a measles-containing vaccine (MCV) 10-dose vial to a 5-dose vial.

In contrast to Indonesia’s in-person approach, Ethiopia’s initial consultation was conducted as a virtual workshop in March 2022, achieving similar objectives with respect to criteria selection and other process design elements. The criteria selected included safety, vaccine price, cost-effectiveness, equity, cold chain considerations, and the total vaccine wastage rate. Among the broad range of stakeholders involved in the consultation were representatives from the EPI program at the Ministry of Health, health system researchers, EPHI’s measles surveillance unit, the HTA team, the Secretary of the Ethiopian NITAG, relevant experts from the WHO country office, and members of academia. The HTA team subsequently led the preparation of evidence statements, informed by a contextualized modeling analysis generating estimates for several selected criteria and outcome measures of interest.

In June 2022, a face-to-face consultation was organized to appraise the prepared evidence statements and the performance matrix. While it was decided during Ethiopia’s first consultation to use quantitative MCDA, as was the case in Indonesia, the EPHI team subsequently preferred a qualitative MCDA to appraise the options. The team selected qualitative MCDA as the data available did not allow for a clear selection of quantitative thresholds for the scale. This meant deliberating the prepared evidence statements and performance matrix but not calculating total scores for each option. The outcomes of the stakeholder deliberations were documented in a final report and presented during a national EPI dissemination workshop in Ethiopia to inform further decision-making by the Ministry of Health.

### 2.3. Documenting Reflections on Enablers and Constraints of CAPACITI Decision-Support Tool Implementation

The reflections shared in this paper comprise a synthesis of critical perspectives by country focal points tasked with overseeing the implementation processes within their respective countries. Reflections are based on feedback shared by participants at the end of the appraisal workshops, deliberations within their respective teams, and in-depth dialogues between the focal points and WHO staff, who contributed specialized technical support.

## 3. Results

### 3.1. Enablers of CAPACITI Decision-Support Tool Implementation

Successful implementation of the CAPACITI decision-support tool was attributed to the commitment of national stakeholders to using innovative practices to facilitate and shape engagement across the health system. The national teams felt supported to implement the innovative practice by the CAPACITI guidance manual in combination with a range of self-study training materials, a draft reporting template, and needs-based training support provided by the WHO.

### 3.2. Constraints of CAPACITI Decision-Support Tool Implementation

There were two constraints affecting the described implementations of the CAPACITI tool that should be noted. First, the COVID-19 pandemic greatly disrupted the process in both countries. In Indonesia, there was a delay in the completion of the process (October 2019–November 2021). In Ethiopia, it was not feasible to organize an initial in-person consultation, leading to attrition of participants during the online format. Moreover, the final consultation in Ethiopia coincided with a COVID-19 vaccination campaign, preventing the participation of several stakeholders with relevant expertise and impacting the resulting buy-in of recommendations. In response to this constraint, the team in Ethiopia adapted their second consultation to pursue a qualitative MCDA approach during the appraisal step, which placed more emphasis on the discussion of the evidence statements and gaps rather than the visual aid and performance matrix.

Second, during evidence collection, both countries noted evidence gaps, particularly for the criterion relating to cost-effectiveness. In Indonesia, the ITAGI decided to generate the needed evidence, conducting cost-effectiveness studies to strengthen the evidence base for their recommendation. This was feasible as they were supported by a dedicated health economics working group with extensive experience in conducting cost-effectiveness studies. Meanwhile, in Ethiopia, such studies were not feasible prior to the final consultation. Hence, one of the outcomes of the deliberation was a request to generate evidence to address this gap.

The different ways in which both countries successfully overcame these implementation constraints highlight the flexibility of the CAPACITI process to respond to various country contexts.

### 3.3. Benefits to the Immunization Program and Broader Health System

The implementation of the structured approach offered by the CAPACITI decision-support tool permitted Ethiopia and Indonesia to strengthen their decision-making by reducing external influence and increasing objectivity. This can yield enhanced buy-in, increased national ownership, and efficiency of the country program. There are three key mechanisms through which this was achieved:

- The approach provided countries with a deliberative and inclusive process that included relevant stakeholders with various functions in the health system and ultimately led to shared and aligned decision-making. The CAPACITI tool structured their discussion across two consultations, which strengthened all stakeholders’ confidence in the decision and improved transparency;
- The MCDA approach encouraged users to consider a broader set of criteria and systematically identify and summarize available evidence when making immunization-related decisions. In Indonesia, criteria ranged from vaccine efficacy to budget impact, alongside other critical factors such as safety and local production. The discussion and agreement among the stakeholders on a set of considered criteria strengthened the overall process and resulting recommendation. Ethiopia appreciated the systematic documentation of the various evidence sources supporting a diverse group of stakeholders to come to a recommendation;
- The stepwise approach helped identify and make explicit important evidence gaps, which can contribute to setting the research agenda, both at national and regional levels, encouraging collaboration between different sectors.

## 4. Discussion

The CAPACITI decision-support tool implementation was enabled by national stakeholder commitment and comprehensive support materials from the WHO but faced constraints from the COVID-19 pandemic and evidence gaps, particularly regarding cost-effectiveness. Despite these challenges, the tool provided significant benefits to immunization programs and health systems in Ethiopia and Indonesia, fostering inclusive decision-making processes, encouraging evidence-based decisions through an MCDA approach, and identifying research priorities.

The CAPACITI decision-support tool led to shared and aligned decision-making in Indonesia and Ethiopia, respectively, which strengthened stakeholder confidence in the decision, improved transparency, and led to more robust decisions. The discussion and agreement among the stakeholders on a set of criteria strengthened the overall process and resulting recommendation, making explicit important evidence gaps. This aligns with the principles outlined in IA2030 [3], which emphasize the importance of involving stakeholders, ensuring transparency, and prioritizing evidence-based decision-making in immunization programs.

The decision-making environment for new vaccine introduction in LMICs is complex given the range of immunization strategies and activities available to countries, combined with economic and financial constraints many LMICs face [9]. Decision-making about new vaccine introductions, including strategic decisions about medium- to short-term priority vaccines for introduction, requires the simultaneous consideration of multiple criteria that collectively capture the broader value of vaccines in terms of benefits and impact across the vaccine life cycle, referred to by WHO as the “full value of vaccines” [9,10]. A recent systematic review of criteria considered in national decision-making for the introduction of new vaccines showed that, indeed, many criteria are considered, noting that programmatic and acceptability aspects were not as often considered and that NITAGs increasingly take into consideration economic evaluations [11]. The Indonesian and Ethiopian experiences with CAPACITI illustrate how, in each country, a structured criteria selection process involving a wide range of national stakeholders supported the identification of a shared and comprehensive set of criteria reflecting the full value of vaccines, including the selection of economic criteria but also programmatic and acceptability criteria.

The aforementioned review and decision-making process showed that the use of models and cost-effectiveness analysis of vaccines to better inform vaccine introduction decisions at the local level requires capacity strengthening via technical assistance, especially in low-capacity settings [11]. The authors of the review further report that standardization of economic evaluation methods and adherence to guidelines should be promoted to allow for improved quality and more straightforward comparison and appraisal of economic models when considered in decision-making. This creates a better understanding of what economic evaluation findings mean and how they should be interpreted. The CAPACITI tool responds to this need by providing detailed guidance on how economic evaluations, including cost-effectiveness and budget impact analysis, can be considered alongside other criteria based on MCDA best-practice guidance [12,13,14,15]. For instance, in Indonesia, the strong and capable health economics working group supporting the NITAG conducted cost-effectiveness and budget impact analysis studies; their subsequent use in decision-making was guided by the CAPACITI steps. Future users of CAPACITI will benefit further from incorporating guidance being developed on translating modeled evidence into decision-making into the tool’s guidance and training materials.

The tool’s future application is particularly promising to support new and underutilized vaccine introduction prioritization, as illustrated by Indonesia. This effectively represents an opportunity to align with WHO’s encouragement of utilizing MCDA as part of the development of National Immunization Strategies. These processes are expected to be led by national authorities to ensure ownership of the process and decisions that are grounded in evidence and established criteria [16]. Decision-makers and groups such as NITAGs can benefit from the support offered by CAPACITI to strengthen their decision-making. The growing pressure on such groups to prioritize warrants that appropriate implementation support be made available [17]. Future real-world applications will support the ongoing refinement and adaptation of tools like CAPACITI based on lessons learned to remain responsive to evolving country needs.

## 5. Conclusions

The implementation of the CAPACITI decision-support tool in Indonesia and Ethiopia benefitted immunization program decision-making through a structured, inclusive process enabling broader health system collaboration. These experiences illustrated the adaptability of the tool to address country-specific needs in a transparent and evidence-based manner accounting for the country context.

Both countries embraced the multi-criteria approach to obtain a holistic and explicit view of the drivers underpinning their prioritization process. National stakeholders welcomed this novel approach for engagement across the immunization program and the health system. While the process was not without some constraints, the available training and guidance were effective in overcoming these. The value expressed by Indonesia and Ethiopia of the decision-support tool developed by WHO confirms the appropriateness of this approach as a response to the increasing call for guidance on the prioritization of health interventions.

## Figures and Tables

**Table 1:** The five steps of the CAPACITI decision-support tool.

Step of the Decision-Support Tool:	Description:
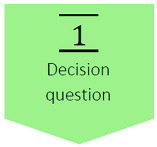	**OBJECTIVES** What recommendation is needed and why? **CONTEXT** What is the current situation? What are the main implications of the recommendation? **SCOPE** Which options will you compare? **PARTICIPATION** Are the right stakeholders involved? Have you got the experts you need?
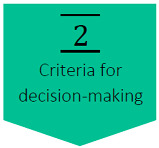	**CRITERIA** Select which criteria will be used. **WEIGHTS** Come to an agreement on whether certain criteria are more important by assigning weights to criteria. **RULES AND SCORING SCALES** Decide how the evidence will be assessed against each of the criteria.
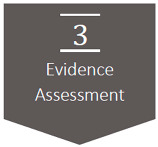	**EVIDENCE STATEMENTS** Write a concise overview of available evidence and its quality for each criterion. **PERFORMANCE MATRIX** Generate a high-level comparison of each option according to the criteria.
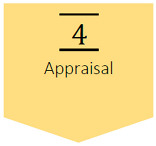	**BY CRITERION** Compare the different options according to each criterion. Are the differences between the options significant? What are the major evidence limitations? **ACROSS CRITERIA** Compare the overall performance of each option. Which option(s) performs better than others overall? Could this change with better data?
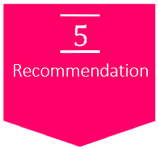	**RECOMMENDATION** What is the final decision? What is the rationale? How strong is the recommendation? **COMMUNICATION AND MONITORING** How will the recommendation be monitored? When will the recommendation next be reviewed? Are there priority data gaps to fill?

**Table 2:** Comparison of how key elements of the CAPACITI decision-support tool were set in Indonesia and Ethiopia and results achieved.

	Indonesia	Ethiopia
**Type of decision**	Vaccine introduction prioritization	Vaccine switch decision
**Context**	New vaccine introduction planning to inform a national multi-year strategic plan for immunization	Optimization of vaccine portfolio to increase coverage for measles vaccine
**Decision-question**	Which rollout scenario for Rotavirus, Human Papillomavirus (HPV), and Pneumococcal Conjugate Vaccine (PCV) do we select for introduction into the Expanded Programme on Immunization (EPI)?	Should we switch from a 10-dose measles-containing vaccine (MCV) vial to a 5-dose MCV vial?
**Criteria for decision-making**	Burden of disease, impact, vaccine availability, adverse events following immunization (AEFI), cold chain capacity, budget impact, community acceptance, schedule, local production, cost-effectiveness, and level of wastage	Safety, vaccine price, cost-effectiveness, equity, cold chain considerations, and the total vaccine wastage rate
**Type of MCDA**	Quantitative MCDA	Qualitative MCDA
**Result**	Prioritized scale-up scenarios for Rotavirus, HPV, and PCV	Recommendation to collect additional evidence on cost-effectiveness
**National team(s) leading the implementation of the CAPACITI decision-support tool**	Indonesian Technical Advisory Group for Immunization (ITAGI) and EPI Ministry of Health (MoH)	Ethiopian Public Health Institute (EPHI)

## Data Availability

Please find the link for downloading the CAPACITI decision-support tool version 2.1 and training materials discussed in this manuscript here: https://www.who.int/teams/immunization-vaccines-and-biologicals/immunization-analysis-and-insights/vaccine-impact-value/economic-assessments/vaccine-prioritization (accessed on 6 January 2024).
